# Dietary diversity and metabolic health among people in Västerbotten, Sweden

**DOI:** 10.1038/s41430-025-01649-3

**Published:** 2025-07-22

**Authors:** Anna Winkvist, Ciara Mangan, Ingegerd Johansson, Annemarie E. Bennett

**Affiliations:** 1 Department of Internal Medicine and Clinical Nutrition, Sahlgrenska Academy, University of Gothenburg, Gothenburg, Sweden; 2 Department of Clinical Medicine, The University of Dublin, Trinity College, Dublin, Ireland; 3 School of Biological, Health and Sport Sciences, Technological University Dublin, Dublin, Ireland; 4 Department of Odontology, Umeå University, Umeå, Sweden

**Keywords:** Epidemiology, Dyslipidaemias

## Abstract

**Background:**

High dietary diversity is recognized as a crucial element of diet quality because this ensures abundant nutrients. A diverse diet may also provide health benefits beyond nutritional adequacy. Few studies have evaluated associations with lipid profile. The aim of this study was to investigate the association between dietary diversity and metabolic health and common measures of diet quality in a large population-based sample.

**Methods:**

This was a cross-sectional analysis within the Västerbotten Intervention Programme (VIP). Participants filled out an extensive health questionnaire including a 66-item semi-quantitative food frequency questionnaire (FFQ). The FFQ was used to create dietary diversity scores (DDS) for 5 major and 13 minor food groups. Associations between DDS and concurrent fasting lipid profile and measured body mass index (BMI) were explored with multivariable linear regression. Correlation analyses were used to explore the relationship between DDS and diet quality.

**Results:**

The mean age of participants was 51 ± 8.3 years, with females comprising 50.8% of the study population (*n* = 82,171). Higher DDS was associated with decreased total cholesterol, low-density lipoprotein cholesterol, high-density lipoprotein cholesterol and triglycerides (all *p* < 0.01). DDS had no significant association with BMI. DDS had a positive correlation with the Relative Mediterranean Diet Score, the Healthy Nordic Food Index and the Healthy Diet Score, and a negative correlation with the Dietary Inflammatory Index (all *p* < 0.001).

**Conclusion:**

The results add to the body of research showing metabolic health benefits of dietary diversity. More consistent methods of measuring dietary diversity should be developed, with careful consideration given to the healthfulness of foods included in the definition.

## Introduction

Dietary diversity has long been a crucial element of high quality diets by ensuring sufficient intake of nutrients essential for the body’s normal physiological functions [1]. However, dietary diversity may extend beyond nutritional adequacy and exert distinct influences on health and hence dietary diversity should not be equated with diet quality [2]. Mechanisms by which dietary diversity may optimise health include more diverse intestinal microbiota [3], which can lead to reduced risk of suboptimal health outcomes such as obesity [4], type 2 diabetes mellitus [5] and inflammatory bowel diseases [6]. Dietary diversity across plant-based foods has also been associated with increased exposure to bioactive compounds such as phytochemicals, vitamins, minerals and fibres which have been shown to improve health [7]. However, the evidence is inconsistent, with some studies reporting that dietary diversity may be associated with poor diet quality and increased food-consumption, which can lead to excess energy intake [8] and suboptimal health outcomes such as obesity [9, 10].

Definitions of dietary diversity vary between countries, and messages on dietary diversity are incorporated into guidelines differently [11]. For example, American dietary guidelines recommend that the population “Choose a variety of options from each food group” [12], whereas Swedish guidelines recommend the inclusion of a variety of plant-based foods rather than variety across all food groups [13]. The diversity of diets has traditionally been assessed through counts, which involves counting the number of foods or food groups consumed within a specified time frame [14]. Food item-based indicators and food group-based indicators are two types of counts. Food item-based indicators assigns points for each food item consumed during a specific period, and food group-based indicators assigns points for each food group consumed over a specific period [15].

The diversity in food supplies has decreased by 70% over the past 50 years [16]. Out of the roughly 7000 crops and several thousand animals utilised by humans for sustenance, only 12 crops and 5 animal species contribute to approximately 75% of the world’s food energy supply [17]. In Europe, approximately 45% of self-reported energy intake comes from the consumption of beef, pork, wheat, and potatoes [18]. The decreasing variety in food supplies and the resulting decline in biodiversity and impairment of ecosystem functions can negatively impact diet quality and the environment [19, 20]. Emergence of contemporary invasive agriculture, which seeks to streamline biological diversity and encourage uniformity to achieve economies of scale, align with the reduction of biodiversity observed [21]. Loss of biodiversity is linked with increased emergence and transmission of infectious disease with harmful impacts on human health as it poses a threat to food security [22]. Loss of biodiversity also puts pressure on single species of foods.

Many studies have evaluated the relationship between dietary diversity and body mass index (BMI) [2, 10, 15, 23–27]. A large systematic review of 16 cross-sectional-observational studies using food group-based indicators reported that 7 studies identified non-significant associations, 5 identified positive associations, and 4 reported inverse associations [23]. Systematic reviews by Mozaffari et al. [2] and Vadiveloo et al. [10] corroborate these findings, also concluding that evidence on the relationship between dietary diversity and obesity is inconsistent, likely depending on the healthfulness of foods included in the analyses.

Less research has been carried out on the relationship between dietary diversity and lipid profile including total cholesterol (TC), low-density lipoprotein cholesterol (LDL-C), high-density lipoprotein cholesterol (HDL-C), and triglycerides. A recent systematic review on associations between food group-based indicators and lipids identified only two studies to include in a meta-analysis [28]. The analyses showed that there was a significant inverse association between dietary diversity scores and triglycerides. No significant associations were found for TC, LDL-C or HDL-C. In sum, the relationship between dietary diversity and metabolic health remains unclear. Further evidence is of importance for human and planetary health. The aim of this project was to evaluate food group based dietary diversity in a large population-based sample of inhabitants in Västerbotten County, Sweden, in relation to concurrent measures of metabolic health and common indicators of diet quality.

## Methods

### Study population

The Västerbotten Intervention Programme (VIP) is an ongoing, population-based prospective study, with dietary intake data from participants of the VIP making up the majority of the Northern Swedish Diet Database [29]. The VIP was established to mitigate the morbidity and mortality associated with cardiovascular disease and diabetes in Västerbotten County [30]. Since 1985, residents have been invited by their local health centre for medical screening and health counselling upon reaching the ages 40, 50 and 60 years, although exact age at the visit may vary somewhat due to routines at the health centres. Participation rate has been 48–67% [31]. Data for the current analyses contain participants who attended their health centre during the years 2000–2016. Only data collected during participants’ first visit during this time-period were included for analysis.

### Dietary assessment and Dietary Diversity Score

Participants filled out a semi-quantitative 66-item Food Frequency Questionnaire (FFQ) on their visit to their local health centre. The FFQ covered the preceding 12-month period [32]. Within the FFQ, participants reported the frequency of intake on a 9-level scale ranging from “never” to “≥4 times per day” [33]. The subjects estimated portion sizes by viewing four pictures depicting progressively larger servings of common foods [33]. Daily intake of macro- and micronutrients was determined using the Swedish national food composition database [34]. The FFQ has been validated using plasma biomarkers [32, 35]. Two food group-based Dietary Diversity Scores (DDS) based on counts were constructed from the 66-items in the FFQ. Reported foods were grouped into 5 major and 13 minor food groups (see Supplementary Tables S1 and S2). One point was assigned for each reported food group (major as well as minor) consumed during the specified period of time. Specific items that were not considered healthy and that did not belong to any of the specified groups such as beverages and edible fats and some mixed dishes such as pizza were excluded. The reference period for intake frequency was established, requiring food items to be consumed at least twice per week to be counted towards the DDS.

### Health Variables

Individuals attended their health centre after an overnight fast [30]. Body weight (measured in kilograms) and height (measured in metres) were measured in light clothing by trained nurses and BMI was calculated as weight (kg)/height (m)² and categorised according to WHO. An oral glucose tolerance test (OGTT) was performed with a 75 g oral glucose load following WHO standards [30]. Fasting blood samples were collected by venepuncture and within 1 h frozen at –20 °C and within 1 week at −80 °C. Total serum cholesterol (TC) and triglycerides were measured at the health centres using a Reflotron bench top analyser (Boerhinger Mannheim GmbH Diagnostica, Baden-Wurttemberg, Germany) until September 9, 2009. After this date, measurements were conducted using an enzymatic routine method at the nearest hospitals’ clinical chemistry department. Values obtained using Reflotron were calibrated to align with values derived from the enzymatic method [33]. During the entire period of the study, only for individuals who were identified as at increased cardiometabolic risk (i.e., elevated triglycerides) were samples sent for more detailed blood lipid analysis [30]. Here, HDL-C was measured and thereafter LDL-C was calculated as: total cholesterol – HDL-C – 0.45 x triglycerides.

### Lifestyle variables

Participants completed a comprehensive questionnaire, providing details on their socioeconomic status, psychological conditions, personal and familial health history, social support and network, work stress, physical activity, sleeping habits, alcohol consumption and tobacco use, and eating habits [30]. Physical activity level, at work and at leisure time was measured using the Cambridge index of Physical Activity, allowing participants to be categorised as, overall inactive, moderately inactive, moderately active, or active [36]. Smoking status was categorised into current smoker, ex-smoker, and never smoker. Education status was divided into basic level, senior high school, and university. Civil status was categorised into unmarried, married/cohabiting, divorced/ separated and widowed.

### Exclusion criteria

Individuals were excluded from analyses if more than 10% of the FFQ data was missing; they did not complete the pictures indicating portion sizes; their food intake level (total energy intake divided by estimated basal metabolic rate) was below the 1st percentile or exceeded the 99th percentile; body weight was missing, or weight was less than 35 kg, height was less than 130 cm or more than 210 cm, or BMI was less than 15 kg/m² or more than 70 kg/m².

### Statistical analysis

Statistical analyses were conducted using IBM SPSS Version 29.01.0. Descriptive categorical data are presented using counts and percentages. Associations between categorical variables were assessed using the Chi-square test with Yates’ Continuity Correction for 2×2 contingency tables. Normally distributed continuous data are presented using means and standard deviations (SD). Multivariable linear regression analysis was used to examine the effect of minor and major food group DDS on the metabolic health outcomes. A crude regression model was run without any potential confounders included. Sex, age, BMI, education level, smoking status, alcohol intake and activity level were thereafter included as potential confounders in the multivariable models, although BMI was excluded as a confounder in the analysis of the relationship between DDS and BMI. For the multivariable analysis of lipid profile, TC values above 15.0 mmol/L or below 0.15 mmol/L, HDL-C values below 0.15 mmol/L or above 7.0 mmol/L and triglyceride values below 0.5 mmol/L or above 20.0 mmol/L were excluded. Regression analyses stratified by sex also were carried out.

Spearman bivariate correlations were used to examine the correlation between major and minor food group DDS and the relative Mediterranean Diet Score (rMDS) as defined by Buckland et al. [37], the Healthy Nordic Food Index (HNFI) as defined by Olsen et al. [38], the Healthy Diet Score (HDS) as defined by Nettleton et al. [39] and the Dietary Inflammatory Index (DII) as defined by Shivappa et al. [40, 41] (Supplementary Table S3). For rMDS, HNFI and HDS, higher numbers are equivalent to healthier diets. For DII, higher numbers are equivalent to more pro-inflammatory diets. Spearman’s rank correlation coefficient was used as the values for rMDS, HNFI, HDS and DII were not normally distributed, as judged using the Kolmogorov-Smirnov test. Significance was taken at *p* < 0.05.

## Results

### Descriptive characteristics of study population

Overall, 82,171 unique VIP participants were included, with 50.8% females and 49.2% males. The mean age (SD) for males and females were 51.2 ± 8.3 and 51.3 ± 8.3 years, respectively (Table 1). The mean BMI for males and females was in the overweight category, however significantly more (*p* < 0.001) females had a healthy BMI when compared with males. Overall, the females’ lipid profile was clinically more favourable than that of the males. LDL-C and triglycerides were significantly higher in males (*p* < 0.001), and HDL-C was significantly higher in females (*p* < 0.001). Of the participants, 2.3% reported having diabetes, with 70% more males than females reporting the condition. A significantly higher proportion of females had a university education when compared to males (40.0% vs. 28.2%, respectively, *p* < 0.001).

**Table 1:** Descriptive characteristics of the Västerbotten Intervention Programme participants *(n* = 82,171).

Variable	Male (*n* = 40,460)	Female (*n* = 41,711)	*p-*value^a^
	*Mean* *±* *SD*	*Mean* *±* *SD*	
Age (years)	51.2 ± 8.3	51.3 ± 8.3	0.29
BMI (kg/m²)	27.1 ± 4.0	26.2 ± 4.9	<0.001
Serum triglycerides (mmol/L)	1.5 ± 1.0	1.2 ± 0.7	<0.001
**Serum cholesterol (mmol/L)**			
Total Cholesterol	5.5 ± 1.1	5.5 ± 1.1	0.038
LDL Cholesterol^b^	3.6 ± 1.0	3.4 ± 1.0	<0.001
HDL Cholesterol^b^	1.3 ± 0.4	1.6 ± 0.5	<0.001
**OGTT (mmol/L)**			
0-h interval	5.6 ± 1.2	5.4 ± 0.9	<0.001
2-h interval	6.4 ± 1.9	6.8 ± 1.7	<0.001
	***n (%)***	***n (%)***	***p-value***^***c***^
**Age group (years)**			0.098
35–44	1,1926 (29.5)	12,102 (29.0)	
45–54	11,964 (29.6)	12,224 (29.3)	
>55	16,567 (40.9)	17,384 (41.7)	
**BMI (kg/m²)**^**d**^			<0.001
Underweight	88 (0.2)	443 (1.1)	
Healthy weight	12,455 (30.8)	19,327 (46.3)	
Overweight	19,985 (49.4)	14,015 (33.6)	
Obese	7932 (19.6)	7926 (19.0)	
**Self-reported diabetes**^**e**^			<0.001
Yes	1158 (2.9)	716 (1.7)	
No	39,101 (97.1)	40,753 (98.3)	
**Education status**			<0.001
Basic level	12,975 (32.1)	10,914 (26.2)	
High school	16,093 (39.8)	14,115 (33.8)	
University	11,392 (28.2)	16,682 (40.0)	
**Civil status**			<0.001
Unmarried	5211 (12.9)	3189 (7.7)	
Married/re-married/co-habiting	31,794 (78.9)	33,323 (80.2)	
Divorced/separated	3019 (7.5)	4032 (9.7)	
Widowed	287 (0.7)	1022 (2.5)	
**Smoking status**			<0.001
Smoker^f^	5905 (14.6)	6299 (15.1)	
Ex-smoker	12,706 (31.4)	13,701 (32.8)	
Never smoker	21,849 (54.0)	21,711 (52.1)	
**Physical activity level**^**g**^			<0.001
Inactive	7226 (17.9)	7129 (17.2)	
Moderately inactive	10,719 (26.6)	11,046 (26.6)	
Moderately active	11,704 (29.0)	11,137 (26.8)	
Active	10,680 (26.5)	12,216 (29.4)	

^a^Comparison between normally distributed continuous data assessed using an Independent Samples t-test.

^b^Only participants with elevated TC had HDL-C measured (males *n* = 28,649 and females *n* = 28,862) and LDL-C calculated (males *n* = 26,871 and females *n* = 27,644).

^c^Comprison between categorical variables assessed using the Chi-squared test with Yates’ Continuity Correction for 2×2 contingency tables.

^d^Categorised according to World Health Organisation classification of BMI.

^e^Includes those with all self-reported subtypes of diabetes.

^f^Includes occasional and daily smokers.

^g^Classified according to Cambridge Activity Index [36].

*SD* standard deviation, *BMI* body mass index, *LDL* low density lipoprotein, *HDL* high density lipoprotein, *OGTT* oral glucose tolerance test.

### Dietary diversity of the study population

Table 2 displays DDS among the whole study population as well as for males and females. Two-thirds (63.3%, *n* = 52,021) of the total cohort included all five major food groups in their diet, with 30% more females than males including all five groups (71.3%, *n* = 29,736 and 55.1%, *n* = 22,285, respectively). Male participants were significantly more likely (*p* < 0.001) to include none of the fruit or vegetable items in their diet. Specifically, 25.0% males included none of the fruit items and 16.2% included none of the vegetable items, compared with 8.8% females including none of the fruit items and 4.8% including none of the vegetable items.

**Table 2:** Dietary diversity characteristics of the VIP population cohort *(n* = 82,171).

Score	*n* food groups	Total *n* (%)	Male n (%)	Female n (%)	*p*-value^a^
Total Diversity: Major Food groups	0–3	8170 (9.9)	6010 (14.8)	2169 (5.1)	<0.001
	4	21,980 (26.7)	12,165 (30.1)	9815 (23.5)	
	5	52,021 (63.3)	22,285 (55.1)	29,736 (71.3)	
Total Diversity: Minor Food Groups	0–6	14,371 (17.6)	9553 (23.6)	4818 (11.5)	<0.001
	7–8	26,434 (32.2)	13,260 (32.8)	13,174 (13.6)	
	9–10	30,851 (37.7)	13,404 (33.1)	17,447 (41.8)	
	11–13	10,515 (12.8)	4243 (10.5)	6272 (15.1)	
Fruit Diversity	0	13,790 (16.8)	10,128 (25.0)	3662 (8.8)	<0.001
	1	26,746 (32.5)	12,606 (31.2)	14,140 (33.9)	
	2	33,165 (40.4)	14,300 (35.3)	18,865 (45.2)	
	3	8470 (10.3)	3426 (8.5)	5044 (12.1)	
Vegetable Diversity	0	8534 (10.4)	6542 (16.2)	1992 (4.8)	<0.001
	1	10,267 (12.5)	6620 (16.4)	3647 (8.7)	
	2	21,503 (26.2)	11,484 (28.4)	10,019 (24.0)	
	3	38,882 (47.2)	14,957 (37.0)	23,865 (57.2)	
	4	3045 (3.7)	857 (2.1)	2188 (5.2)	
Dairy Diversity	0–2	11,935 (14.5)	18,277 (45.2)	15,757 (37.8)	<0.001
	3	26,143 (31.8)	12,461 (30.8)	13,682 (32.8)	
	4	15,762 (19.2)	6922 (17.1)	8840 (21.2)	
	5–8	6261 (7.6)	2800 (6.9)	3432 (8.1)	
Protein Diversity	0	14,968 (18.2)	7438 (18.4)	7548 (18.1)	<0.001
	1	21,875 (26.6)	10,526 (26.0)	11,349 (27.2)	
	2	19,575 (23.8)	9560 (23.6)	10,015 (24.0)	
	3–4	19,403 (23.6)	9602 (23.7)	9801 (23.5)	
	5–14	6350 (7.8)	3334 (8.2)	2998 (7.2)	
Carbohydrate Diversity	0–3	21,574 (26.2)	10,688 (26.4)	10,886 (26.1)	<0.001
	4	17,760 (21.6)	8682 (21.5)	9078 (21.8)	
	5	17,647 (21.5)	8455 (20.9)	9192 (22.0)	
	6–7	20,582 (25.1)	10,151 (25.1)	10,431 (25.0)	
	8–12	4608 (5.7)	2484 (6.2)	2124 (5.1)	

^a^Association between categorical variables assessed using the Chi-square test with Yates’ Continuity Correction for 2×2 contingency tables.

### Association of dietary diversity with lipid profile

Greater dietary diversity across major and minor food groups were significantly associated with reduced TC, LDL-C, HDL-C and triglyceride levels, when confounders were adjusted for (Table 3). However, when stratified by sex, the negative association between DDS and HDL-C was only significant among males (Supplementary Tables S4 and S5).

**Table 3:** Multivariable linear regression analysis exploring the associations between dietary diversity and lipid profile among 82,027 participants in the VIP study.

	Major Food Groups			Minor Food Groups		
	Beta coefficient^a^ ± SEM	*p*-value	95% CI	Beta coefficient ± SEM	*p*-value	95% CI
TC crude	−0.032 ± 0.005	<0.001	(−0.042, −0.022)	−0.008 ± 0.002	<0.001	(−0.011, −0.004)
TC adjusted^b^	−0.050 ± 0.005	<0.001	(−0.060, −0.040)	−0.012 ± 0.002	<0.001	(−0.016, −0.009)
LDL-C^c^ crude	−0.037 ± 0.006	<0.001	(−0.049, −0.026)	−0.012 ± 0.002	<0.001	(−0.017, −0.008)
LDL-C^c^ adjusted^b^	−0.025 ± 0.006	<0.001	(−0.037, −0.013)	−0.007 ± 0.002	0.002	(−0.011, −0.030)
HDL-C^d^ crude	0.048 + 0.002	<0.001	(0.043, 0.053)	0.017 + 0.001	<0.001	(0.015, 0.019)
HDL-C^d^ adjusted^b^	−0.010 ± 0.002	<0.001	(−0.015, −0.005)	−0.003 ± 0.001	0.002	(−0.005, −0.001)
TG^e^ crude	−0.086 ± 0.004	<0.001	(−0.094, −0.078)	−0.029 ± 0.002	<0.001	(−0.032, −0.026)
TG^e^ adjusted^b^	−0.022 ± 0.004	<0.001	(−0.030, −0.015)	−0.006 ± 0.002	<0.001	(−0.009, −0.003)

^a^Beta coefficients express change in lipids (mmol/L) per increase one unit in diet diversity.

^b^Adjusted for sex, age, BMI, physical activity, smoking status, and education status.

^c^LDL-C calculated for a population of *n* = 54,515.

^d^HDL-C measured among a population of *n* = 57,348.

^e^TG measured among a population of *n* = 81,826.

*SEM* standard error of the mean, *CI* confidence interval, *TC* total cholesterol, *LDL-C* low density lipoprotein cholesterol, *HDL-C* high density lipoprotein cholesterol, *TG* triglycerides, *BMI* body mass index.

### Association of dietary diversity with BMI

In an unadjusted model, a significant (*p* < 0.001) negative association was found between major and minor food group dietary diversity and BMI (Table 4); however, in the multivariable model the association was no longer significant (*p* > 0.05). No differences in these associations between males and females were noted (Supplementary Tables S5 and S6).

**Table 4:** Multivariable linear regression analysis of relationship between dietary diversity and BMI (*n* = 82,171).

	Major Food Groups			Minor Food Groups		
	Beta coefficient ± SEM	*p*-value	95% CI	Beta coefficient ± SEM	*p*-value	95% CI
BMI crude	−0.238 + 0.021	<0.001	(−0.280, −0.196)	−0.039 + 0.008	<0.001	(−0.109, −0.078)
BMI adjusted^a^	−0.026 + 0.008	0.022	(−0.069, 0.017)	−0.013 + 0.008	0.118	(−0.028, 0.003)

^a^Adjusted for sex, age, BMI, physical activity, smoking status and, education status.

*SEM* standard error of the mean, *CI* confidence interval, *BMI* body mass index.

### Correlations of dietary diversity with diet quality scores

There was a weak positive correlation between major and minor food group DDS and the rMDS and HDS (Table 5). There was a strong positive correlation between minor food group DDS and the HNFI and, a moderate positive correlation between major food group DDS and the HNFI. There was a strong negative correlation between minor food group DDS and the DII and a moderate negative correlation between major food group DDS and the DII. All *p*-values were <0.001.

**Table 5:** Correlations among dietary diversity scores and diet quality scores among the VIP study population (*n* = 82,171).

Diet Quality Scores	Major Food Groups DDS	Minor Food Groups DDS
*rMDS*	*rho* = 0.199 *p* < *0.001*	*rho* = 0.243 *p* < *0.001*
*HNFI*	*rho* = 0.401 *p* < *0.001*	*rho* = 0.540 *p* < *0.001*
*HDS*	*rho* = 0.243 *p* < *0.001*	*rho* = 0.255 *p* < *0.001*
*DII*	*rho* = *−*0.401 *p* < *0.001*	*rho* = *−*0.556 *p* < *0.001*

*DDS* Dietary Diversity Score, *rMDS* Relative Mediterranean Diet Score, *HNFI* Healthy Nordic Food Index, *HDS* Healthy Diet Score, *DII* Dietary Inflammatory Index.

## Discussion

This study demonstrated a significant association between increased dietary diversity and decreased TC, LDL-C, HDL-C, and triglycerides among inhabitants in Västerbotten, Sweden. No significant association was found between dietary diversity and BMI. Significant correlations between dietary diversity and the diet quality scores were shown. Findings also revealed that females consumed a wider range of fruits, vegetables and dairy, while diversity among protein and carbohydrate food groups were more similar across sexes.

Our finding for triglycerides reflects previous research which reports consistent inverse associations between dietary diversity and triglycerides. As mentioned above, the meta-analysis on associations between DDS and lipid profile published in 2021 identified two studies to include and reported a significant inverse association with triglycerides but no other significant associations [28]. Both included studies were cross-sectional, conducted in Iran and had small sample sizes (581 and 160, respectively) [42, 43]. The study by Azadbakht et al. showed significant inverse associations between dietary diversity among the whole grains food group and triglyceride levels, and among vegetables and TC and LDL-C [42]. The study by Farhangi et al. [43] included adults with metabolic syndrome and revealed significant inverse associations between dietary diversity and triglycerides. However, no associations were identified with TC, LDL-C or HDL-C [43]. Differences between findings in that and the current study may be explained by different sample sizes, number of food groups included and the health profile of the population.

A few additional studies on dietary diversity and lipid profile exist, that were excluded from the Qorbani et al. meta-analysis [28]. Gholizadeh et al. [44] conducted another cross-sectional study in Iran, where 150 pre-diabetic patients and 150 healthy subjects were included. Higher DDS was significantly associated with lower triglycerides and higher HDL-C. Tian et al. [45] evaluated similar associations among 4308 adults in the China Health and Nutrition Survey. Surprisingly, higher DDS was significantly associated with higher triglycerides and lower HDL-C in males but not in females. Finally, Kant et al. [46] analyzed data from NHANES among 8719 disease-free Americans. They demonstrated significant inverse association between DDS and total cholesterol, but no associations with LDL-C, HLD-C or triglycerides.

An unexpected finding of ours was that increased dietary diversity was significantly associated with decreased HDL-C in the adjusted model, although with significantly increased HDL-C in the crude model. Perhaps this is a chance finding as it is in opposition to the beneficial effect of dietary diversity on the other serum lipids and only found among males. Also, HDL-C was only measured on a subsample of the VIP population with cardiometabolic risk factors, opening for selection bias and less robust model estimates. Still, the same association only among males was found in the Chinese study by Tian et al. [45]. Perhaps greater variety of some less healthy foods could have a negative effect on HDL-C and this could be investigated in research on diet diversity within the food groups. More research is needed to explore this unexpected association.

The present study is the first to find a protective effect of increased dietary diversity on TC, LDL-C and triglycerides, although with a negative effect on HDL-C among males. However, the wider literature on this association is minimal, with many methodological differences between studies. More research, specifically longitudinal prospective studies, are required to explain the biological associations between dietary diversity and lipid profile as this has not been elucidated in existing research.

We did not find any significant association between dietary diversity and BMI. Existing research is here inconsistent, with a systemic review by Salehi-Abargouei et al. identifying 7 studies reporting a non-significant association between dietary diversity and BMI, 5 studies reporting a positive association, and 4 studies reporting an inverse association [23]. The inconsistent results could be attributed to differences in methods of measuring dietary diversity, what food items were included, and reference periods used [2]. We made efforts to exclude less healthy foods while keeping a large breadth of food items. This led to the inclusion of some food items which may not be optimal for health (see Supplementary Tables S1 and S2). The current study used a population-based sample in Sweden and a self-reported FFQ, whereas others have used a population living in Hong Kong and FFQ interview [25] and women in low socioeconomic areas of Brazil and 24-h recall [27]. These differences may contribute to the inconsistencies in the literature.

There was a positive correlation between DDS among minor and major food groups and the rMDS, the HDS and the HNFI, while there was a negative correlation with the DII. This result was expected as the food items included in the minor and major food groups in this study are primarily healthy foods and this is also indicated by higher numbers for the rMDS, the HFI and the HDS [37–39]. The DII focuses on pro- and anti-inflammatory properties of foods, including more of a mix of what foods are considered healthy and unhealthy, and where higher positive numbers are associated with more pro-inflammatory diets [40]. Foods such as coffee and alcohol here are classified as anti-inflammatory, with negative numbers.

Strengths of our study include its population-based design with a large sample size. High quality methodology was used, with highly trained nurses collecting the data, including a validated FFQ, measured weight and height and fasting serum samples for lipid profile measurements. Some limitations are also acknowledged. As in all research, participation bias is possible. The study design is cross-sectional, meaning that reverse causality cannot be ruled out. The use of the self-reported FFQ is susceptible to under-reporting intake of unhealthy foods and/or over-reporting intake of healthy foods. The FFQ was not designed to optimally capture dietary diversity, which led to the exclusion of many food items due to their lack of healthfulness. Also, the limited number of items in the FFQ (66 items) limits the ability to capture variation within food groups. While this study was population-based, capturing the dietary intake of the Västerbotten population in northern Sweden, a study including a more heterogeneous population may capture more dietary diversity and therefore yield stronger associations with health outcomes.

In conclusion, the present study found that increased dietary diversity had a significant association with concurrent decreased TC, LDL-C, triglycerides and HDL-C, but no significant association with BMI. This study further found that DDS had a significant positive correlation with three diet quality scores: the rMDS, the HNFI and the HDS and a significant inverse correlation with the DII. These results indicate that more research is needed into the association between dietary diversity and metabolic health and to determine the biological reasons for these associations. More consistent methods of measuring dietary diversity should be developed, with careful consideration given to the healthfulness of foods included in the evaluation of dietary diversity. The addition of well-designed studies to this area will contribute beneficially to nutritional epidemiology and have benefits for population health as a result.

## Supplementary information

Dietary diversity and metabolic health among people in Västerbotten, Sweden

## Data Availability

Access to individual-level data can be provided for research purposes but is restricted by laws regarding the privacy of research participants and are therefore not made publicly available. Requests for data can be sent to the Section of Biobank and Registry Support at Umeå University (contact: info.brs@umu.se).
