# Supplementary material for: Dietary diversity and metabolic health among people in Västerbotten, Sweden
Source: Eur J Clin Nutr. 2025 Jul 22;79(10):1046–52. doi: 10.1038/s41430-025-01649-3 (PMC12537496; doi:10.1038/s41430-025-01649-3)
Supplement: Supplementary file 1 — Dietary diversity and metabolic health among people in Västerbotten, Sweden [file 41430_2025_1649_MOESM1_ESM.docx]

Supplement

**Title:** Dietary diversity and metabolic health among people in Västerbotten, Sweden

**Journal:** European Journal of Nutrition

**Authors:** Anna Winkvist^1*^, Ciara Mangan^1,2^, Ingegerd Johansson^3^, Annemarie Bennett^2^

^1^Department of Internal Medicine and Clinical Nutrition, Sahlgrenska Academy, University of Gothenburg, Gothenburg, Sweden

^2^Technological University Dublin and The University of Dublin, Trinity College, Dublin, Ireland

^3^Department of Odontology, Umeå University, Umeå, Sweden

**Corresponding author:** Anna Winkvist Department of Internal Medicine and Clinical Nutrition, Sahlgrenska Academy, University of Gothenburg, Box 459, S 405 30 Gothenburg, Sweden. E-mail [anna.winkvist@nutrition.gu.se](mailto:anna.winkvist@nutrition.gu.se), phone +46-70-6616217

**Supplementary Table S1**. Major food groups (5 groups) created from the Food Frequency Questionnaire

| **Major Food Group** | **Food Items** |
| --- | --- |
| Vegetables | 1. Root vegetables, carrots 2. Tomato, cucumber 3. Lettuce, Chinese cabbage, spinach, kale 4. Brown peas, pea soup |
| Fruit | 1. Berries, fresh or deep frozen 2. Apples, pears, peaches, citrus fruits 3. Banana |
| Meat, fish and egg | 1. Sausage/liverwurst as sandwich topping 2. Meat as sandwich topping 3. Ground meat dishes 4. Beef stew 5. Steak, pork chop 6. Bacon, pork belly, ham 7. Sausage as a main course 8. Poultry 9. Lean fish (perch, cod) 10. Fatty fish (herring, whitefish, salmon) 11. Salted fish (herring) 12. Smoked fish/meat 13. Egg, egg dishes |
| Dairy | 1. Cream, sour cream 2. Hard cheese, 28% fat 3. Hard cheese, 10-17% fat 4. Soured milk, yoghurt, 3% fat 5. Soured milk, yoghurt, 0,5% fat 6. Milk, 0,5% fat 7. Milk, 1,5% fat 8. Milk, 3% fat |
| Carbohydrates | 1. Dark crisp bread, e.g., rye 2. Wholegrain bread 3. White bread including crisp/flat bread 4. Wheat buns, rusk 5. Oatmeal, graham-, rye- or barley porridge 6. Fibre-rich cereals, e.g. musli 7. Cornflakes, Special K, etc 8. Potato, boiled or baked 9. Fried potatoes, French fries 10. Rice 11. Spaghetti, macaroni 12. Pancakes, waffles, potato dumplings, boiled |
| Foods from food frequency questionnaire not included in 5 major food groups | 1. Diluted fruit syrup drinks 2. Drip-filtered coffee 3. Boiled, unfiltered coffee 4. Tea 5. Beer, 2.8% alcohol 6. Beer, 2.8 – 3.5% alcohol 7. Beer, 4.5% alcohol 8. Wine 9. Spirits, 40% alcohol 10. Water 11. Ice cream 12. Sweets, e.g. chocolate, candy 13. Sugar cubes, sugar, honey, marmalade jam 14. Cakes, cookies, pastry 15. Chips, popcorn, salted nuts, etc 16. Pizza 17. Hamburger 18. Butter (80% fat on sandwich) 19. Butter on sandwich 20. Margarine, 40-60% fat, on sandwich 21. Margarine 80% fat, on sandwich 22. Butter in cooking 23. Margarine in cooking 24. Oil in cooking 25. Vinaigrette 26. Rose hip soup, fruit syrup soup, thickened fruit syrup and water |

**Supplementary Table S2.** Minor food groups (13 groups) created from the Food Frequency Questionnaire

| **Minor Food Group** | **Food Items** |
| --- | --- |
| Reduced fat dairy | 1. Hard cheese, 10-17% fat 2. Soured milk, yoghurt, 0.5% fat 3. Milk, 0.5% fat 4. Milk, 1.5% fat |
| Full fat dairy | 1. Milk, 3% 2. Soured milk, yoghurt, 3% fat 3. Hard cheese 28%, fat 4. Cream, sour cream |
| Berries | 1. Berries, fresh or deep frozen |
| Other fruit | 1. Banana 2. Apples, pears, peaches, citrus fruits |
| Root vegetable | 1. Root vegetables, carrots |
| Dark green leafy | 1. Lettuce, Chinese cabbage, spinach, kale |
| Other vegetables | 1. Tomato, cucumber 2. Brown bean, pea soup |
| Flesh meat – red (including processed) | 1. Sausage/liver wurst as sandwich topping 2. Beef stew 3. Steak, pork chop 4. Bacon, pork belly, ham 5. Sausage as a main course 6. Ground meat dishes |
| Poultry and egg | 1. Poultry 2. Eggs/egg dishes |
| Fish | 1. Lean fish (perch, cod) 2. Fatty fish (herring, whitefish, salmon) 3. Salted fish (herring) |
| Whole grains | 1. Dark crisp bread, e.g., rye 2. Wholegrain bread 3. Oatmeal, graham-, rye- or barley porridge 4. Fibre-rich cereals, e.g. muesli |
| Non whole grains | 1. White bread including crisp/flat bread 2. Wheat buns, rusk – check this 3. Cornflakes/Special K |
| Other/starchy carbohydrates | 1. Potato, boiled or baked 2. Fried potatoes, French fries 3. Rice 4. Spaghetti, macaroni 5. Pancakes, waffles, potato dumplings, boiled |
| Foods from food frequency questionnaire not included in minor food groups | 1. Ice cream 2. Sweets, e.g. chocolate, candy 3. Sugar cubes, sugar, honey, marmalade jam 4. Cakes, cookies, pastry 5. Chips, popcorn, salted nuts, etc 6. Diluted fruit syrup drinks 7. Oil in cooking 8. Vinaigrette 9. Rose hip soup, fruit syrup soup, thickened fruit syrup and water 10. Drip-filtered coffee 11. Boiled, unfiltered coffee 12. Tea 13. Beer, 2.8% alcohol 14. Beer, 2.8 – 3.5% alcohol 15. Beer, 4.5% alcohol 16. Wine 17. Spirits, 40% alcohol 18. Water 19. Hamburger 20. Pizza 21. Meat as sandwich topping 22. Smoked fish/meat 23. Butter (80% fat on sandwich) 24. Butter on sandwich 25. Margarine, 40-60% fat, on sandwich 26. Margarine 80% fat, on sandwich 27. Butter in cooking 28. Margarine in cooking |

**Supplementary Table S3.** Diet Quality Indices

Relative Mediterranean Diet Score (rMDS)

A relative Mediterranean Diet Score (rMDS) was calculated as previously described by Buckland et al (37). The score captures adherence to a Mediterranean style diet. Tertiles of intake, expressed as g*1000/kcal*day, were calculated for intake of nine components: vegetables excluding potatoes; fruit including nuts and seeds; legumes, fresh and frozen fish excluding fish products and preserved fish, olive oil and cereals. The tertiles were assigned values of 0-2. For total meat and dairy products, similar tertiles were constructed and the scoring was reversed to account for a possible negative effect on health. Alcohol was scored 2 for moderate consumption and 0 for consumption outside of this range. The final score had a maximum of 9, indicating high adherence to a healthy Mediterranean-style diet.

Healthy Nordic Food Index (HNFI)

The HNFI was calculated as previously described by Olsen et al. (38). It captured intake of six components: fish, cabbages, whole grain rye, whole grain oats, apples and pears, and root vegetables. For each component, individuals received a score of 0 or 1 based on whether he or she placed below or above the relevant sex-specific median for that component. A participant’s total score for the HNFI could range from 0 to 6 points, with 6 indicating high adherence to a healthy Nordic style diet.

Healthy Diet Score (HDS)

A Healthy Diet Score (HDS) was calculated as previously described by Nettleton et al (39). The score is based on intake of eight components. Favorable components include fish, fruit (except juice), vegetables (except potatoes) and whole grain. Unfavorable components include red and processed meat, desserts and sweets, sugar-sweetened beverages and fried potatoes. Within each sex, intakes are ranked in ascending quartile ranks for favorable groups and in descending quartile ranks for unfavorable groups. The sum of the quartile ranks yields the score, with a maximum of 24 and higher scores reflecting adherence to a healthier diet.

Dietary Inflammatory Index (DII)

A Dietary Inflammatory Index (DII) was calculated as previously described by Shivappa et al (40, 41). First, individual intake of 30 food parameters were expressed as z-scores relative to global intake data. Thereafter, estimated inflammatory contributions of each food parameter were calculated, based on literature-derived overall inflammatory effect-values. Food parameters known to contribute to inflammation yield positive values and food parameters known to prevent inflammation yield negative values. Finally, contributions from all consumed food parameters were summed to a final Dietary Inflammatory Index, with low values representing anti-inflammatory actions and high values representing pro-inflammatory actions.

**Supplementary Table S4.** Multivariable linear regression analysis of association between major food dietary diversity and lipid profile, stratified by sex (*n*=82,027)

ᵃAdjusted for sex, age, BMI, physical activity, smoking status, and education status

^b^LDL-C calculated for a population of *n*=54,515

^c^HDL-C measured among a population of *n=*57,511

^d^TG measured among a population of *n=*81,826

Abbreviations: SEM, standard error of the mean, CI, confidence interval, TC, total cholesterol, LDL-C, low density lipoprotein cholesterol, HDL-C, high density lipoprotein cholesterol, TG, triglycerides

|  | **Male** | | | **Female** | | | | |
| --- | --- | --- | --- | --- | --- | --- | --- | --- |
|  | **Beta coefficient + SEM** | ***p*-value** | **95% CI** | | **Beta coefficient**  **+SEM** | ***p*-value** | **95% CI** |  |
| TC crude | -0.037+0.007 | <0.001 | (-0.062, -0.036) | | -0.007+0.009 | 0.448 | (-0.024, 0.011) |  |
| TC adjustedᵃ | -0.049+0.007 | <0.001 | (-0.062, -0.036) | | -0.038+0.008 | <0.001 | (-0.055, -0.022) |  |
| LDL-C^b^ crude | -0.018+0.008 | 0.003 | (-0.038, -0.008) | | 0.001+0.010 | 0.893 | (-0.019, 0.022) |  |
| LDL-C^b^ adjustedᵃ | -0.023+0.008 | 0.003 | (-0.038, -0.008) | | -0.027+0.010 | 0.007 | (-0.046, -0.007) |  |
| HDL-C^c^ crude | 0.006+0.003 | 0.291 | (-0.002, 0.008) | | 0.021+0.004 | <0.001 | (0.007, 0.024) |  |
| HDL-C^c^ adjustedᵃ | -0.011+0.003 | <0.001 | (-0.017, -0.005) | | -0.006+0.004 | 0.185 | (-0.014, 0.003) |  |
| TG^d^ crude | -0.048+0.006 | <0.001 | (-0.060, -0.036) | | -0.037+0.005 | <0.001 | (-0.047, -0.026) |  |
| TG^d^ adjustedᵃ | -0.020+0.006 | <0.001 | (-0.031, -0.008) | | -0.022+0.005 | <0.001 | (-0.032, -0.012) |  |
|  |  |  |  | |  |  |  |  |

**Supplementary Table S5.** Multivariable linear regression analysis of association between minor food dietary diversity and lipid profile, stratified by sex *(n*=81,027)

|  | **Male** | | | **Female** | | |
| --- | --- | --- | --- | --- | --- | --- |
|  | **Beta coefficient + SEM** | ***p*-value** | **95% CI** | **Beta coefficient + SEM** | ***p*-value** | **95% CI** |
| TC crude | -0.013+0.003 | <0.001 | (-0.018, -0.008) | -0.001+0.003 | 0.636 | (-0.007, 0.004) |
| TC adjustedᵃ | -0.013+0.003 | <0.001 | (-0.018, -0.007) | -0.009+0.003 | <0.001 | (-0.015, -0.004) |
| LDL-C^b^ crude | -0.006+0.003 | 0.046 | (-0.012, 0.000) | -0.004+0.003 | 0.266 | (-0.010, 0.003) |
| LDL-C^b^ adjustedᵃ | -0.005+0.003 | 0.084 | (-0.011, 0.001) | -0.008+0.003 | 0.009 | (-0.015, -0.002) |
| HDL-C^c^ crude | 0.001+0.001 | 0.527 | (-0.002, 0.003) | 0.006+0.001 | <0.001 | (0.004, 0.009) |
| HDL-C^c^ adjustedᵃ | -0.004+0.001 | <0.001 | (-0.007, -0.002) | -0.001+0.001 | 0.715 | (-0.003, 0.002) |
| TG^d^ crude | -0.019+0.002 | <0.001 | (-0.024, -0.014) | -0.011+0.002 | <0.001 | (-0.015, -0.007) |
| TG^d^ adjustedᵃ | -0.007+0.002 | 0.003 | (-0.012, -0.002) | -0.004+0.002 | 0.012 | (-0.008, -0.001) |

ᵃAdjusted for sex, age, BMI, physical activity, smoking status, and education status

^b^LDL-C calculated for a population of *n*=54,515

^c^HDL-C measured among a population of *n=*57,511

^d^TG measured among a population of *n=*81,826

Abbreviations: SEM, standard error of the mean, CI, confidence interval, TC, total cholesterol, LDL-C, low density lipoprotein cholesterol, HDL-C, high density lipoprotein cholesterol, TG, triglycerides

**Supplementary Table S5** Multivariable linear regression analysis of association between major food dietary diversity and BMI, stratified by sex (*n*=82,171)

|  | **Male** | | | **Female** | | |
| --- | --- | --- | --- | --- | --- | --- |
|  | **Beta coefficient + SEM** | ***p*-value** | **95% CI** | **Beta coefficient + SEM** | ***p*-value** | **95% CI** |
| BMI crude | -0.118+0.024 | <0.001 | (-0.165, -0.070) | -0.120+0.040 | 0.003 | (-0.197, -0.042) |
| BMI adjustedᵃ | -0.019+0.024 | 0.427 | (-0.067, 0.028) | -0.030+0.040 | 0.455 | (-0.108, 0.048) |

ᵃAdjusted for age, physical activity, smoking status and, education status

Abbreviations: SEM, standard error of the mean, CI, confidence interval, BMI, body mass index

**Supplementary Table S6,** Multivariable linear regression analysis of association between minor food dietary diversity and BMI, stratified by sex (*n*=82,171.)

|  | **Male** | | | **Female** | | |
| --- | --- | --- | --- | --- | --- | --- |
|  | **Beta coefficient + SEM** | ***p*-value** | **95% CI** | **Beta coefficient + SEM** | ***p-*value** | **95% CI** |
| BMI crude | -0.058+0.009 | <0.001 | (-0.076, -0.039) | -0.049+0.013 | <0.001 | (-0.075, -0.023) |
| BMI adjustedᵃ | -0.015+0.010 | 0.110 | (-0.034, 0.003) | -0.008+0.013 | 0.536 | (-0.034, 0.018) |

ᵃAdjusted for, age, physical activity, smoking status and, education status

Abbreviations: SEM, standard error of the mean, CI, confidence interval, BMI, body mass index
